# Historical Analysis of the Brazilian Cervical Cancer Screening Program from 2006 to 2013: A Time for Reflection

**DOI:** 10.1371/journal.pone.0138945

**Published:** 2015-09-24

**Authors:** Ricardo Filipe Alves Costa, Adhemar Longatto-Filho, Céline Pinheiro, Luiz Carlos Zeferino, José Humberto Fregnani

**Affiliations:** 1 Graduate Program of Oncology, Barretos Cancer Hospital, Barretos, São Paulo, Brazil; 2 Barretos School of Health Sciences Dr. Paulo Prata – FACISB, Barretos, São Paulo, Brazil; 3 Research and Teaching Institute, Barretos Cancer Hospital, Barretos, São Paulo, Brazil; 4 Molecular Oncology Research Center, Barretos Cancer Hospital, Barretos, São Paulo, Brazil; 5 Laboratory of Medical Investigation (LIM 14), Faculty of Medicine, São Paulo University, FMUSP, São Paulo, Brazil; 6 Life and Health Sciences Research Institute, ICVS, School of Health Sciences, Minho University, Braga, Portugal; 7 ICVS/3B’s - PT Government Associate Laboratory, Braga/Guimarães, Portugal; 8 School of Medical Sciences, Women’s Hospital CAISM, Unicamp, Campinas, São Paulo, Brazil; State University of Maringá/Universidade Estadual de Maringá, BRAZIL

## Abstract

**Background:**

The Cervical Cancer Database of the Brazilian National Health Service (SISCOLO) contains information regarding all cervical cytological tests and, if properly explored, can be used as a tool for monitoring and managing the cervical cancer screening program. The aim of this study was to perform a historical analysis of the cervical cancer screening program in Brazil from 2006 to 2013.

**Material and Methods:**

The data necessary to calculate quality indicators were obtained from the SISCOLO, a Brazilian health system tool. Joinpoint analysis was used to calculate the annual percentage change.

**Results:**

We observed important trends showing decreased rates of low-grade squamous intraepithelial lesions (LSIL) and high-grade squamous intraepithelial lesions (HSIL) and an increased rate of rejected exams from 2009 to 2013. The index of positivity was maintained at levels below those indicated by international standards; very low frequencies of unsatisfactory cases were observed over the study period, which partially contradicts the low rate of positive cases. The number of positive cytological diagnoses was below that expected, considering that developed countries with low frequencies of cervical cancer detect more lesions annually.

**Conclusions:**

The evolution of indicators from 2006 to 2013 suggests that actions must be taken to improve the effectiveness of cervical cancer control in Brazil.

## Introduction

Cervical cancer is the fourth most commonly diagnosed cancer in women worldwide, with an estimated 528,000 new cases annually, and it is the fourth most common cause of cancer deaths in women, with 266,000 estimated deaths annually. More than 85% of new cervical cancer cases and more than 87% of the deaths from cervical cancer occur in developing countries [1].

In Brazil, cervical cancer, excluding non-melanomas, is the third most common cancer in women, with an estimated incidence of 15.33 cases per 100,000 women. Cervical cancer is also the third most common cause of cancer deaths among women in Brazil [2].

The lower incidence and mortality of cervical cancer in developed countries can be explained by well-structured cytological screening programs and better medical infrastructure, which are not frequently found in low- and middle-income countries [3].

The Brazilian Ministry of Health recognized the need for a national program for cervical cancer control, and its coordination is the responsibility of the Brazilian National Cancer Institute (INCA) [4]. Brazilian health authorities recommend cytology-based screening at three-year intervals for women between 25 and 64 years of age who have initiated sexual activity. In recent years, multiple approaches to develop an effective cervical screening program have been implemented in Brazil. In 2005, the Action Plan for the Control of Cervical and Breast Cancer proposed six strategic guidelines: increased coverage of the target population, laboratory quality assurance, strengthening of the information system, professional training development, social mobilization strategies and research development [4]. In 2012, to improve the quality and reliability of cytopathological exams, a Quality Management Manual for Cytopathology Laboratory was published by the Brazilian National Institute of Cancer and the Ministry of Health. This manual presents some important indicators for the monitoring of laboratory results, which assess overall and individual performance [5]. The Department of Informatics of the Public Health System (DATASUS) created the Information System of Cervical Cancer Screening (SISCOLO), which contains information regarding all Papanicolaou (Pap) tests collected in the public health system. The SISCOLO was implemented for the management and monitoring of the cervical cancer screening program [4] and is publicly available at http://www2.datasus.gov.br/DATASUS/index.php.

Although the SISCOLO was implemented for monitoring and assisting in the management of the above-mentioned program, this data resource has been poorly exploited and deserves more attention. As a result, the present work is a historical analysis of the cervical cancer screening program quality indicators in Brazil from 2006 to 2013 based on data collected from the SISCOLO.

## Materials and Methods

This is a time series study of the cervical cancer screening program quality indicators in Brazil. Data regarding cytopathological exams (n = 81,322,700) were collected from the SISCOLO according to the location of collection (Brazilian state) and age of the women who voluntarily participated in the opportunistic Governmental Brazilian program of cervical cancer prevention from 2006 to 2013. Data regarding the female population was obtained from the DATASUS from 2006 to 2012. All data collected from public access databases (SISCOLO and DATASUS) are anonymous. This study was approved by the Ethics Committee of the Barretos Cancer Hospital (identification: CAAE 26354114.0.0000.5437).

The following quality indicators were determined for women of 25 to 64 years of age: (1) productivity rate; (2) percentage of exams performed during the target age (25–64 years); (3) positivity index (PI); (4) percentage of unsatisfactory exams; (5) percentage of rejected exams; (6) ASC-US (atypical squamous cells of undetermined significance) percentage; (7) ASC-H (atypical squamous cells—high grade) percentage; (8) LSIL (low-grade squamous intraepithelial lesion) percentage; (9) HSIL (high-grade squamous intraepithelial lesion) percentage; (10) ASC (atypical squamous cell) percentage; (11) ASC/abnormal exam rate; and (12) ASC/SIL ratio.

The transformation zone (TZ) percentage, i.e., the percentage of scrubs with representation of the transformation zone, was calculated for all women. This indicator was calculated for women aged < 50 years and women aged ≥ 50 years.

The formulas used to calculate these indicators are presented in Table 1.

**Table 1:** Formulas to calculate quality indicators and reference values.

Indicators		Calculation	Reference Values
**General**	**Productivity rate** ^(a)^	$\frac{\text{number of exams performed }(25\;-\;64)}{\text{number of women }(25\;-\;64)}\times 100$	(a) Number of women unavailable for 2013
	**% Exams Performed in the target age**	$\frac{\text{number of exams performed }(25\;-\;64)}{\text{number of exams performed }(\text{all ages})}\times 100$	Not available
**Pre-analytical**	**% Unsatisfactory**	$\frac{\text{number of unsatisfactory exams }}{\text{number of exams performed }(25\;-\;64)}\times 100$	1% (Average of the collected exams in Brazil in 2010)
	**% Rejected**	$\frac{\text{number of rejected exams }}{\text{number of exams performed }(25\;-\;64)}\times 100$	0.1% (Average of the collected exams in Brazil in 2010)
	**% TZ (≥50 years)**	$\frac{\text{number of TZ exams }(\geq 50)}{\text{number of satisfactory exams }(\geq 50)}\times 100$	50%
	**% TZ (<50 years)**	$\frac{\text{number of TZ exams }(<50)}{\text{number of satisfactory exams }(<50)}\times 100$	68%
**Analytical**	**% Positivity Index**	$\frac{\text{number of abnormal exams }(25\;-\;64)}{\text{number of satisfactory exams }(25\;-\;64)}\times 100$	3–10%
	**% ASC-US**	$\frac{\text{number of ASC-US exams }(25\;-\;64)}{\text{number of satisfactory exams }(25\;-\;64)}\times 100$	Not available
	**% ASC-H**	$\frac{\text{number of ASC-H exams }(25\;-\;64)}{\text{number of satisfactory exams }(25\;-\;64)}\times 100$	Not available
	**% LSIL**	$\frac{\text{number of LSIL exams }(25\;-\;64)}{\text{number of satisfactory exams }(25\;-\;64)}\times 100$	Not available
	**% HSIL**	$\frac{\text{number of HSIL exams }(25\;-\;64)}{\text{number of satisfactory exams }(25\;-\;64)}\times 100$	0 5–1 0% (USA, 0.5%; Canada, 0.6%; UK, 1.1%; Norway, 1.1%)
	**% ASC**	$\frac{\text{number of ASC exams }(25\;-\;64)}{\text{number of satisfactory exams }(25\;-\;64)}\times 100$	<4–5%
	**ASC/Abnormal rate**	$\frac{\text{number of ASC exams }(25\;-\;64)}{\text{number of abnormal exams }(25\;-\;64)}\times 100$	<60%
	**ASC/SIL ratio**	$\frac{\text{number of ASC exams }(25\;-\;64)}{\text{number of SIL exams }(25\;-\;64)}\times \text{100}$	<3

TZ, transformation zone; ASC-US, atypical squamous cells of undetermined significance; ASC-H, atypical squamous cells cannot exclude high-grade squamous intraepithelial lesion; ASC, atypical squamous cells; LSIL, low-grade squamous intraepithelial lesion; HSIL, high-grade squamous intraepithelial lesion; SIL, squamous intraepithelial lesion.

^(a)^ Number of women unavailable for 2013.

### Processing of data and statistical analysis

All data were stored in.csv format, which allowed for management of the data using R software (The R Foundations for Statistical Computing) and Microsoft Excel 2010 (Microsoft Corporation 2010). R software and Microsoft excel were used to organize the large volume of data from the DATASUS and allowed for the creation of new spreadsheets.

The annual percentage change (APC) for each indicator was calculated using the Joinpoint Regression Program Version 4.1.1 (August 2014; Statistical Methodology and Applications Branch, Surveillance Research Program, National Cancer Institute). Joinpoint analysis allows for interpretation of changes over time to determine whether these changes are statistically significant. Joinpoint software allows for adjustment of data series using a minimum number of inflection points (zero, in which case the trend is represented by a single line segment) and tests whether the inclusion of more inflection points (joinpoints) in the model has statistical significance. To test for significance, the software uses the Monte Carlo permutations method, and to determine the APC, the software uses the natural logarithm of the rates calculated using the following formulas: y = mx+b, y = ln(rate) and x = calendar year, and APC = 100x(e^m^-1). The APC and estimates of the trend are used, with calendar year as the regression variable. Each significant point indicates an increase or decrease in the rate [6]. To describe the linear trend for each period, the APC values with 95% confidence intervals (95% CIs) are calculated for each trend.

## Results

From 2006 to 2013, 81,322,750 cytopathological exams were performed in Brazil, with 62,397,698 (76.7%) women within the screening target age range from 25–64 years.

**Fig 1:**
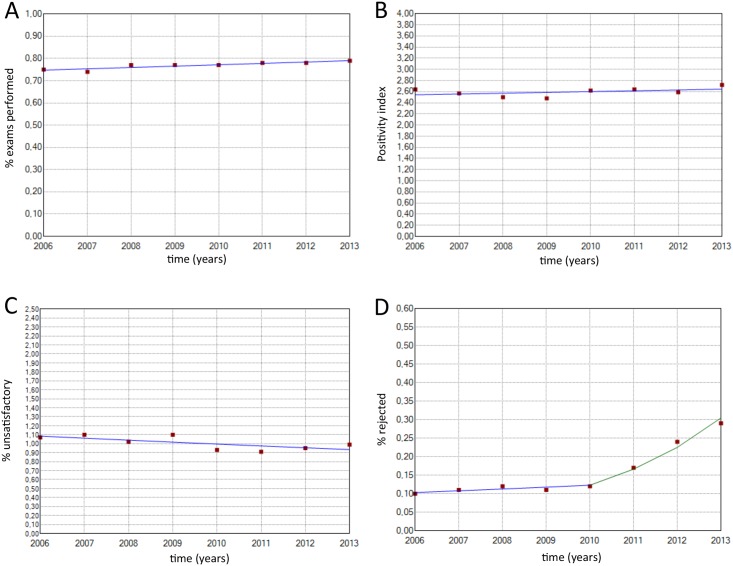
Historical description of the following indicators. (A) % exams performed (2006–2013: APC = -0.7 CI 95%: -2.1; 1.2). (B) % positivity index (2006–2013: APC = 0.56 CI 95%: -0.5; 1.7). (C) % unsatisfactory (2006–2013: APC = -2.1 CI 95%: -4.3; 0.1). (D) % rejected (2006–2010: APC = 4.5 CI 95%: -5.1; 15.0) (2010–2013: APC = 35.4 CI 95%: 16.3; 56.6).

**Fig 2:**
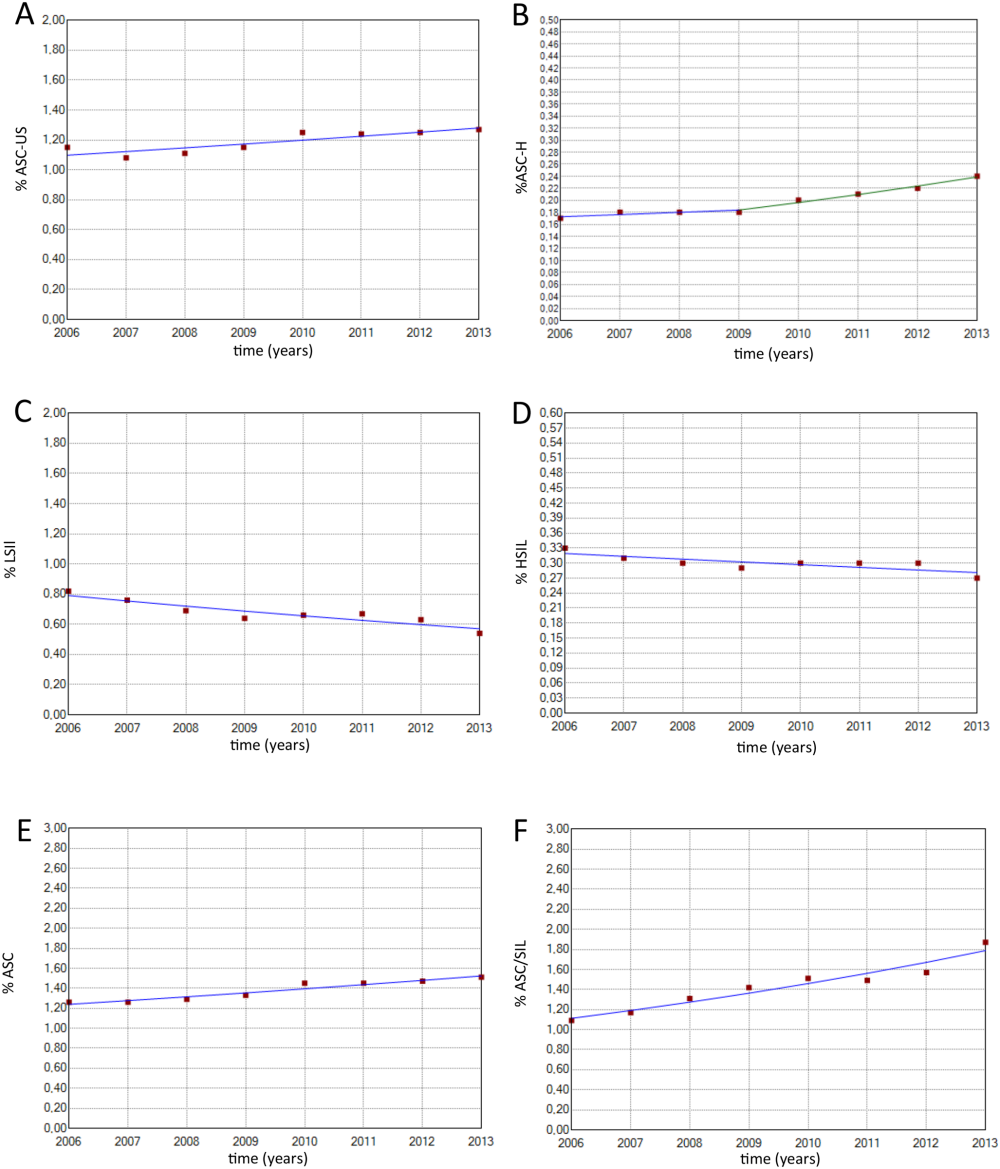
Historical description of the following indicators. (A) % ASC-US (2006–2013: APC = 2.2 CI 95%: 0.9; 3.5). (B) % ASC-H (2006–2009: APC = 2.1 CI 95%: -2.0; 6.4) (2009–2013: APC = 6.8 CI95%: 4.0; 9.6). (C) % LSIL (2006–2013: APC = -4.6 CI 95%: -6.6; -2.5). (D) % HSIL (2006–2013: APC = -1.8 CI 95%: -3.2; -0.6). (E) % ASC (2006–2013: APC = 3.0 CI 95%: 2.1; 3.8). (F) ASC/SIL (2006–2013: APC = 7.0 CI 95%: 5.2; 8.9).

Table 2 shows the distribution of exams according to Pap test results and age. Table 3 shows the values for each indicator, and Table 4 shows the corresponding APC values. Figs 1 and 2 show the main results.

**Table 2:** Number of exams according to Pap Smear results and age for Brazil 2006–2013.

Exams	<25 years	25–64 years	> 64 years	Total
**Performed**	14,477,595	62,397,698	4,447,457	81,322,750
**Satisfactory**	14,307,468	61,668,690	4,372,053	80,348,211
**Unsatisfactory**	147,852	638,872	69,630	856,354
**Rejected**	22,336	94,162	6,527	123,025
**Abnormal**	545,726	1,596,740	126,390	2,269,856
**TZ** ^**(a)**^	8,854,751	36,321,788	1,749,901	46,926,440
**ASC-US**	223,742	731,692	57,083	1,012,517
**ASC-H** ^**(a)**^	17,416	115,732	14,726	147,874
**ASC**	241,158	847,424	71,809	1,110,391
**LSIL**	252,928	418,715	14,092	685,735
**HSIL**	26,144	185,695	16,912	228,751
**SIL**	279,072	604,410	31,004	914,486
**AGC**	11,153	83,557	5,826	100,572
**AIS**	18	359	33	410
**SCC/ADC**	1,138	15,366	5,963	22,467

TZ, transformation zone; ASC-US, atypical squamous cells of undetermined significance; ASC-H, atypical squamous cells cannot exclude high-grade squamous intraepithelial lesion; ASC, atypical squamous cells; LSIL, low-grade squamous intraepithelial lesion; HSIL, high-grade squamous intraepithelial lesion; SIL, squamous intraepithelial lesion; AGC, atypical glandular cells; AIS, adenocarcinoma in situ; SSC, squamous cell carcinoma; ADC, adenocarcinoma.

^(a)^ Data collected from June 2006.

**Table 3:** Values of the cervical cancer screening program quality indicators in Brazil 2006–2013.

Indicators	Year							
	2006	2007	2008	2009	2010	2011	2012	2013
**Productivity rate (%)**	15.98	16.72	16.72	17.32	16.61	15.97	15.51	-
**% Exams Performed**	74.69	73.65	76.62	77.00	77.48	77.96	78.36	78.69
**% Unsatisfactory**	1.07	1.10	1.02	1.10	0.93	0.91	0.95	0.99
**% Rejected**	0.10	0.11	0.12	0.11	0.12	0.17	0.24	0.29
**% TZ (≥50 years)**	54.11	52.85	49.18	47.13	46.00	45.12	45.71	46.03
**% TZ (<50 years)**	66.83	67.74	66.92	65.37	65.12	64.07	64.91	64.94
**% Positivity Index**	2.64	2.57	2.50	2.48	2.62	2.64	2.59	2.72
**% ASC-US**	1.15	1.08	1.11	1.15	1.25	1.24	1.25	1.27
**% ASC-H**	0.17	0.18	0.18	0.18	0.20	0.21	0.22	0.24
**% LSIL**	0.82	0.76	0.69	0.64	0.66	0.67	0.63	0.54
**% HSIL**	0.33	0.31	0.30	0.29	0.30	0.30	0.30	0.27
**% ASC**	1.26	1.26	1.29	1.33	1.45	1.45	1.47	1.51
**ASC/Abnormal rate (%)**	47.77	49.02	51.78	53.48	55.19	54.77	56.58	55.52
**ASC/SIL ratio**	1.09	1.17	1.31	1.42	1.51	1.49	1.57	1.87

TZ, transformation zone; ASC-US, atypical squamous cells of undetermined significance; ASC-H, atypical squamous cells cannot exclude high-grade squamous intraepithelial lesion; ASC, atypical squamous cells; LSIL, low-grade squamous intraepithelial lesion; HSIL, high-grade squamous intraepithelial lesion; SIL, squamous intraepithelial lesion.

**Table 4:** APC values of quality indicators for Brazil 2006–2013.

Indicators	Trend 1				Trend 2			
	Period	Annual percent change	CI 95%		Period	Annual percent change	CI 95%	
		(APC)	LL	UL		(APC)	LL	UL
**Productivity rate (%)**	2006–2009	2.4	-1.8	6.7	2009–2012	-3.6	-7.6	0.5
**% Exams Performed**	2006–2013	0.8 *	0.4	1.2				
**% Unsatisfactory**	2006–2013	-2.1	-4.3	0.1				
**% Rejected**	2006–2010	4.5	-5.1	15.0	2010–2013	35.4 *	16.3	56.6
**% TZ (> = 50 years)**	2006–2010	-4.4 *	-6.0	-2.8	2010–2013	0.4	-2.2	3.0
**% TZ (<50 years)**	2006–2013	-0.7 *	-1.1	-0.2				
**% Positivity Index**	2006–2013	0.6	-0.5	1.7				
**% ASC-US**	2006–2013	2.2 *	0.9	3.5				
**% ASC-H**	2006–2009	2.1	-2.0	6.4	2009–2013	6.8 *	4.0	9.6
**% LSIL**	2006–2013	-4.6 *	-6.6	-2.5				
**% HSIL**	2006–2013	-1.8 *	-3.2	-0.6				
**% ASC**	2006–2013	3.0 *	2.1	3.8				
**ASC/Abnormal rate (%)**	2006–2010	3.8 *	1.7	5.8	2010–2013	0.4	-2.7	3.6
**ASC/SIL ratio**	2006–2013	7.0 *	5.2	8.9				

TZ, transformation zone; ASC-US, atypical squamous cells of undetermined significance; ASC-H, atypical squamous cells cannot exclude high-grade squamous intraepithelial lesion; ASC, atypical squamous cells; LSIL, low-grade squamous intraepithelial lesion; HSIL, high-grade squamous intraepithelial lesion; SIL, squamous intraepithelial lesion; APC, annual percent change; CI, Confidence Interval; LL, Lower Limit; UP, Upper Limit.

* The APC is significantly different from 0 (*p*-value<0.05).

The productivity rate, positivity index and percentage of unsatisfactory exams remained relatively stable over the entire study period. A yearly significant increase was observed in the percentage of exams performed on women in the target age range (APC = 0.8). Additionally, yearly significant increases in ASC-US percentage (APC = 2.2), ASC percentage (APC = 3.0) and ASC/SIL ratio (APC = 7.0) and a yearly significant decrease in the percentage of LSIL (APC = -4.6), percentage of HSIL (APC = -1.8) and percentage of TZ were observed in women aged <50 years (APC = -0.7).

Finally, four indicators of quality showed one inflection point, indicating a change in the trend; these indicators included the percentage of rejected exams, ASC-H percentage, rate of ASC in abnormal exams and percentage of TZ in woman aged ≥ 50 years. The percentage of rejected exams significantly increased (APC = 34.4) from 2010 to 2013; prior to 2010, this rate was relatively stable. The ASC-H percentage remained stable until 2009 and then increased significantly (APC = 6.8) until 2013. The ASC/abnormal exam rate increased significantly (APC = 3.8) from 2006 to 2010, after which time it remained stable, and the percentage of TZ in women aged ≥50 years significantly decreased from 2006 to 2010 and then remained stable.

## Discussion

The SISCOLO is an important tool for improving the Brazilian opportunistic cervical cancer program, as it contains a significant amount of data regarding Pap smear tests that can be used to calculate quality indicators. Through these data, it is possible to identify fragilities and strengths and to evaluate indicators to adjust the course of action. However, the SISCOLO information has not yet been used at its maximum potential because it does not allow for the identification of women or the calculation of the actual number of women who effectively underwent Pap screening. In fact, the SISCOLO only provides the overall number of tests that were performed. In addition, the SISCOLO data are only for women under the National Health System (SUS) and do not cover women who use supplementary health services [7]. Indeed, when collecting information from the SISCOLO, we noticed that some data were incomplete (e.g., 2013 data from Amapá state), and this may have been the result of the lack of a well-established flow of information between institutions. In order to overcome some of the above-mentioned limitations, the Brazilian Ministry of Health is implementing the Cancer Information System (SISCAN) web platform, which integrates the information systems of cervical (SISCOLO) and breast (SISMAMA) cancer screening programs. SISCAN will be associated with the National Health Registry and will comprise a module to identify and convene women register in the SUS, to carry out screening tests according to the periodicity and recommended age [8].

Focusing on the general results, we observed that the productivity rate, which is the ratio of the number of Pap tests and the number of women in the target age range (25–64 years), remained stable over the study period. If we consider the three-year interval of screening based on the recommendation of the Brazilian guidelines, we observed a cumulative productivity rate of approximately 45%. However, this number cannot be used as the real coverage rate, as a significant number of women with a normal Pap test underwent screening more than once in a three-year period [9, 10], possibly due to the overuse of Pap smears by physicians and a lack of women’s knowledge of Pap test periodicity [11, 12]. In addition, approximately 25% of the population had private health insurance [13], and the percentage of women who underwent a Pap test at private health insurance laboratories is not included in the SISCOLO system. This simple estimation showed that the Pap test coverage rate in Brazil is below 70%. The results presented herein do not support those found in the Brazilian National Household Sample Survey (PNAD) performed in 2008, which reported a coverage rate higher than 80% over a three-year period. PNAD is a research system that uses nationwide household data to produce basic information to study the socioeconomic development of the country [14]. However, if we consider that the productivity rate is an overestimation of the coverage rate and that we are counting the number of exams rather than the number of women who underwent a Pap test, the coverage rate appears to be lower than 70%.

Based on the percentage of exams performed on women in the target age range, the goal of the cancer screening program was achieved, as there was a significant increase in this value over the study period. However, approximately 25% of tests are performed on women outside the target age range, which implies the existence of unnecessary financial resource consumption. This high frequency of unnecessary exams outside the target population is frequently observed in opportunistic programs [15–17], as in Brazil.

The analysis of the pre-analytical Pap test results showed that the percentage of unsatisfactory exams did not vary over the study period, and importantly, this indicator follows the World Health Organization (WHO) recommendation for unsatisfactory exams (<5%). However, the percentage of rejected tests significantly increased in recent years (2010–2013). This result can be explained by the lack of care in handling, transportation and identification of samples, which underscores the importance of focusing not only on sample collection but also on all of the steps between sample collection and sample analysis. Another important pre-analytical indicator is the presence of epithelial cells from the transformation zone (TZ), as the presence of these cells increases the likelihood of lesion identification, and the majority of lesions begin to develop in this region [18–20]. Although the results showed that the percentage of TZ cells was close to the Brazilian National Cancer Institute reference value, it significantly decreased over the years, which may be a consequence of problems related to sample collection.

Regarding cytological alterations, the positivity indexes, which indicate the prevalence of cell alteration and characterize the sensitivity of the screening process for detecting lesions, were below those recommended by the Brazilian guidelines (3–10%) [5]. It is concerning that these indexes are significantly below the indexes observed in developed countries, which have already achieved control of cervical cancer incidence, such as Norway (4.9%) [21], the USA (6.8%) [22] and Great Britain (6.5%) [23]. Interestingly, we observed a reduction in LSIL and HSIL detection. The observed HSIL value was approximately 0.3% per year, which is below the expected range (0.5–1.0%) [5] and below the values reported in developed countries, such as the USA and Canada (0.5% and 0.6%, respectively) [24]. In addition, intraepithelial lesion (LSIL and HSIL) detection rates significantly decreased over the study period, which may be explained as a result of a complex network of events that could have limited the quality of cytology-based screening. For example, prior to analyzing the results of Pap exams, events such as inappropriate sample collection and slide preparation are likely to occur. Additionally, difficulty in the detection of intraepithelial lesions by cytotechnologists and/or cytopathologists can occur as a consequence of suboptimal slide preparation, high workload routine, limitations related to the continued education of the professionals or even inadequate training of the professionals. Conversely, this decrease may also be explained by a real reduction in the prevalence of intraepithelial lesions in the population; however, the high ASC-US and ASC-H percentages, which have been found to be increasing, support the first hypothesis of problems in the collection phase and/or the interpretation of the samples. This result highlights the need for better training of the professionals who collect and analyze these samples.

In general, the pre-analytical and analytical phase indicators suggest a suboptimal Pap test performance, which could be attributed to the low professional efficiency in the collection, preparation and reading of the samples. To maintain an adequate level of competence, it is important that the laboratory processes a minimum number of samples per year. In this context, the Pan American Health Organization (PAHO) considers it essential that a laboratory process at least 15,000 exams annually [25]. However, according to a study conducted in Brazil in 2002 on 739 participating laboratories, only 18.9% of the laboratories had performed at least 15,000 exams/year [26], suggesting that the majority of laboratories may be working with examiners who have not adequately developed their skills.

Even laboratories that perform a large amount of exams can show poor indicators due to excessive overload. As elegantly reported by Renshaw and colleagues, the reading of more than 50 slides per day (6 hours of work) initiates an ascendant curve of progressive errors related to fatigue and loss of attention [27]. This variable is far from being controlled in many Brazilian laboratories. In fact, the low price of Pap tests paid by the Government (circa US $2.0) pushes cytotechnicians to examine more than the recommended 50 slides per day. This fact generates a pernicious circle resulting in cytotechnicians getting paid less to analyze more slides than recommended.

Despite the above-mentioned limitations of the current screening program, it is important to note that the increase in access to Pap tests has been associated with a decrease in cervical cancer-related mortality, which has been observed in developed regions in Brazil (South, Southeast and Midwest). However, this success is only partial, as the underdeveloped regions (North and Northeast) have experienced an increase in cervical cancer-related mortality [28].

Based on the findings of the present study and according to the optimal quality control measures, the government should invest more funds in internal quality control during both the pre-analytical phase, via review of the adequacy of collection, fixation and transportation of samples to the laboratory, and the analytical phase, via the training of cytotechnologists to improve their professional skills. Additionally, as some studies show the importance of external quality control of laboratories in the improvement of professionals’ skills in detecting cervical cancer precursor lesions, a systematic improved external control should be implemented at all cytopathology laboratories that provide services to the public health system. The current external system of quality control implemented by the Brazilian Government is limited to a small number of samples in selected laboratories examined periodically [29, 30], which may result in an unrealistic panorama of the laboratories’ performances.

Importantly, one should also keep in mind the recent breakthrough in cervical cancer prevention due to the introduction of human papillomavirus (HPV) vaccines. This event promises to modify the burden of cervical cancer incidence and mortality. However, as the vaccines only protect against oncogenic HPVs, which are responsible for approximately 70% of all cervical cancers, it is important to increase the sensitivity and reproducibility of the screening program. In this regard, HPV DNA testing combined or followed by Pap smear test in HPV-positive cases is as an attractive approach for cervical cancer screening in the present scenario [31].
